# Retraction: Fe(iii) and Cr(vi) ions' removal using AgNPs/GO/chitosan nanocomposite as an adsorbent for wastewater treatment

**DOI:** 10.1039/d6ra90109c

**Published:** 2026-08-20

**Authors:** Abeer El Shahawy, Mahmoud F. Mubarak, Merna El Shafie, Hesham M. Abdulla

**Affiliations:** a Department of Civil Engineering, Faculty of Engineering, Suez Canal University PO Box 41522 Ismailia Egypt abeer_shahawi@eng.suez.edu.eg; b Petroleum Applications Department, Egyptian Petroleum Research Institute (EPRI) Nasr City Cairo 11727 Egypt fathy8753@epri.sci.eg; c Faculty of Science, Mansoura University Mansoura Egypt; d Department of Civil Engineering, Faculty of Engineering, Suez Canal University PO Box 41522 Ismailia Egypt mernaelshafie24@gmail.com; e Botany Dept., Faculty of Science, Suez Canal University Box 41522 Ismailia Egypt hesham_abdulla@science.suez.edu.eg

## Abstract

Retraction of ‘Fe(iii) and Cr(vi) ions' removal using AgNPs/GO/chitosan nanocomposite as an adsorbent for wastewater treatment’ by Abeer El Shahawy *et al.*, *RSC Adv.*, 2022, **12**, 17065–17084, https://doi.org/10.1039/d2ra01612e.

The Royal Society of Chemistry hereby wholly retracts this *RSC Advances* article due to concerns with the reliability of the data.

Concerns were raised with the integrity of the FTIR in Fig. 2, the XRD in Fig. 8 and BET isotherm in Fig. 9. The authors have not been able to provide the raw data.

Given the significance of these concerns, the editor has lost confidence that the findings presented in this paper are reliable.

Hesham Abdulla clarifies that their role was limited to supervising the wastewater analysis conducted at the Suez Canal University Center for Environmental Studies and Consultation, and they were not responsible for generating, processing, validating, interpreting, or retaining the material-characterization data that are the subject of the concerns raised by the journal, including the FTIR, XRD, and BET data presented in Fig. 2, 8, and 9.

The authors were informed about the retraction of the article but have not indicated whether they agree with the decision to retract.

Signed: Laura Fisher, Executive Editor, *RSC Advances*

Date: 13th August 2026

